# Restricted Proliferation During Neurogenesis Contributes to Regionalisation of the Amphioxus Nervous System

**DOI:** 10.3389/fnins.2022.812223

**Published:** 2022-03-24

**Authors:** Giacomo Gattoni, Toby G. R. Andrews, Èlia Benito-Gutiérrez

**Affiliations:** Department of Zoology, University of Cambridge, Cambridge, United Kingdom

**Keywords:** amphioxus, brain development, proliferation, neurogenesis, HCR, EdU pulse-chase, axial progenitors, chordate evolution

## Abstract

The central nervous system of the cephalochordate amphioxus consists of a dorsal neural tube with an anterior brain. Two decades of gene expression analyses in developing amphioxus embryos have shown that, despite apparent morphological simplicity, the amphioxus neural tube is highly regionalised at the molecular level. However, little is known about the morphogenetic mechanisms regulating the spatiotemporal emergence of cell types at distinct sites of the neural axis and how their arrangements contribute to the overall neural architecture. In vertebrates, proliferation is key to provide appropriate cell numbers of specific types to particular areas of the nervous system as development proceeds, but in amphioxus proliferation has never been studied at this level of detail, nor in the specific context of neurogenesis. Here, we describe the dynamics of cell division during the formation of the central nervous system in amphioxus embryos, and identify specific regions of the nervous system that depend on proliferation of neuronal precursors at precise time-points for their maturation. By labelling proliferating cells *in vivo* at specific time points in development, and inhibiting cell division during neurulation, we demonstrate that localised proliferation in the anterior cerebral vesicle is required to establish the full cell type repertoire of the frontal eye complex and the putative hypothalamic region of the amphioxus brain, while posterior proliferating progenitors, which were found here to derive from the dorsal lip of the blastopore, contribute to elongation of the caudal floor plate. Between these proliferative domains, we find that trunk nervous system differentiation is independent from cell division, in which proliferation decreases during neurulation and resumes at the early larval stage. Taken together, our results highlight the importance of proliferation as a tightly controlled mechanism for shaping and regionalising the amphioxus neural axis during development, by addition of new cells fated to particular types, or by influencing tissue geometry.

## Introduction

Vertebrate evolution has led to the origin of nervous systems of striking morphological diversity and functional complexity. Whilst is generally agreed that vertebrate nervous systems are very similar at early developmental stages and probably built up on a same common ground plan, little is known about the mechanisms that makes them architecturally so different later in development (Nieuwenhuys, 2017). Central to this question is understanding the principles governing the establishment of the nervous system “bauplan” (or ground plan), common to all invertebrate and vertebrate chordates, for which amphioxus is ideally placed given its key phylogenetic position, branching at the root of the chordate tree (Garcia-Fernàndez and Benito-Gutiérrez, 2009; Benito-Gutiérrez, 2011; Holland, 2016; Holland and Holland, 2021).

The amphioxus central nervous system (CNS) has a relatively simple morphology compared to that of vertebrates. It consists of a dorsal neural tube, devoid of overt segmentation, with an anteriorly enlarged region that corresponds to the brain and is usually regarded as the “cerebral vesicle.” Despite this apparent simplicity, accumulating gene expression data over the years has revealed a high degree of molecular regionalisation in the amphioxus CNS (Benito-Gutiérrez, 2006; Yu et al., 2007; Holland, 2017), and has identified a diversity of cell types which, albeit in low numbers, collectively represent regions of homology to specific areas of the vertebrate brain and spinal cord (Albuixech-Crespo et al., 2017; Benito-Gutiérrez et al., 2021). Diversity of cell types in the amphioxus brain is also supported by exquisitely detailed descriptions of cell morphologies in late amphioxus larva and adult brains using electron microscopy (EM) (Lacalli et al., 1994; Lacalli, 1996; Wicht and Lacalli, 2005). A great effort is being made to match these cellular morphologies to the reported gene expression patterns (Benito-Gutiérrez et al., 2021; Bozzo et al., 2021; Lacalli, 2021). However, these studies have been performed at very different developmental stages, when there are marked differences in neural tube size and shape, making this exercise very difficult. To fully exploit this knowledge and understand brain regionalisation at a molecular and morphological levels, is therefore necessary to gain insights into the morphogenetic processes regulating the numbers and spatial distribution of individual cells during nervous system development in amphioxus.

In vertebrate embryos, the initially single-layered neuroepithelium is progressively patterned by intrinsic and extrinsic cues that drive its cells into specific cellular behaviours and specialised modes of cell division (Temple and Shen, 2013). These specialised modes of cell division are key to confer tissue shape and to diversify the cell type repertoire. Depending on the division mode, progenitors can either divide to increase cell numbers in specific populations, to augment the progenitor pool, or to produce neuroblasts that differentiate into different types of neurons (Caviness et al., 1995; Götz and Huttner, 2005; Huttner and Kosodo, 2005). In the spinal cord, for example, posterior axial progenitors clonally expand to enlarge the progenitor pool and contribute with new cells to the elongating neural tube (Wilson et al., 2009; Steventon and Martinez Arias, 2017). Here, cell division is required to define form, while in the vertebrate telencephalon asymmetric divisions are key to layer the cortex, as well as to influence neuronal migratory routes and to diversify the neuronal type repertoire (Noctor et al., 2004). A proper balance between proliferation and programmed cell death is key to establish final neuronal numbers, adult brain size, and a proper geometry (Rakic, 1995; Chenn and Walsh, 2002; Ando et al., 2019). Neuronal birth dating studies in vertebrates have also shown that the time at which neurons are born can be predictive of their connectivity and projections, meaning that this temporal control in cell division might be critical to set the right cytoarchitecture (Allaway et al., 2020).

In amphioxus, previous studies have reported spatially restricted proliferation in the anterior tip of the neural tube and the posterior floor plate at the mid-neurula stage (Holland and Holland, 2006). However, the origin, dynamics, and fate of these proliferating cells, and therefore their contributions to the emergence of neural complexity in amphioxus, remain to be investigated. In this work, we present a detailed characterisation of cell division dynamics during early neural development in amphioxus. In doing so, we identify critical roles of proliferation in the diversification of the neural type repertoire, and the regulation of neural tube geometry. We first expand on previous data (Holland and Holland, 2006; Andrews et al., 2021) by constructing a quantitative proliferation landscape during early neural development, which shows that cell division restricts to distinct mitotic niches in the cerebral vesicle and chordoneural hinge at the onset of axial segmentation and elongation. By arresting cell division pharmacologically and using gene expression profiling we birth date and track specific neurons in the anterior cerebral vesicle, demonstrating that cell division is essential to increase the cell type repertoire within the maturing brain. In the posterior neural tube, we show that cell division is necessary to elongate the posterior floor plate and notochord, acting in axial progenitor populations arising from the dorsal blastopore lip of the gastrula. Collectively, our data shows that cell division has multiple roles during amphioxus nervous system development, each reminiscent of vertebrate dynamics. Together, these data suggest that modulation of cell division dynamics might have been a major strategy for the emergence of neural complexity in chordate evolution.

## Materials and Methods

### Animal Husbandry and Embryo Fixation

Adults of the European amphioxus Branchiostoma *lanceolatum* were collected in Banyuls-sur-Mer, France, and transported to Cambridge, United Kingdom, where they were maintained and spawned in a custom-made facility as described in Benito-Gutiérrez et al. (2013). After *in vitro* fertilisation, embryos were raised in filtered artificial salt water (ASW) at 21°C and fixed in ice-cold 3.7% Paraformaldehyde (PFA) + 3-(*N*-morpholino)propanesulfonic acid (MOPS) buffer for 12 h, then washed in sodium phosphate buffer saline (NPBS), dehydrated and stored in 100% methanol (MeOH) at –20°C. Embryonic stage was determined unbiasedly by counting the number of somites. For early stages where somites are not yet formed, we used hours post-fertilisation (hpf) to determine stages.

### EdU Labelling and Detection

For EdU pulse analyses, EdU was applied to live embryos in filtered seawater at a final concentration of 20 μM for 2 h prior to fixation. For EdU pulse-chase analyses, EdU was removed after 2 h of exposure *in vivo* by transferring embryos to a fine 15 μm filter and washing them in an excess of filtered sea water. They were then transferred to a fresh petri dish and incubated until the desired developmental stage.

Fluorescent detection of incorporated EdU was performed following the manufacturer’s instructions, using a Click-it EdU Alexa Fluor 647 Imaging Kit (Invitrogen, Waltham, MA, United States) prior to primary antibody incubation. As advised for enhanced signal, the copper reagent was replenished after 15 min.

To construct proliferation landscapes, Euclidean distances were calculated between each EdU + and/or PhH3 + nucleus and the anterior tip of the cerebral vesicle. Distance values were then normalised to total neural tube length (defined here defined as the distance between the anterior tip of the cerebral vesicle and the chordoneural hinge of the tailbud), and scaled to mean neural tube length across the population. The frequency of nuclei was then calculated in 20 μm bins of the anteroposterior axis, and normalised to embryo number. Normalised values were then plotted for each somite stage using the *ggridges* package in R.

### Pharmacological Perturbation With Hydroxyurea

Live amphioxus embryos were treated with 2 μM hydroxyurea (Sigma, H8627) or an equal volume of dimethylsulfoxide (DMSO; Sigma, 276855). This was performed either between the cup-shaped gastrula and 14-somite stages (8 hpf to 34 hpf at 21°C), or the six-somite and the 12- and 14-somite stages (18 hpf to either 30 or 34 hpf at 21°C).

### Immunohistochemistry

Rehydrated embryos were permeabilised overnight in PBS + 1% DMSO + 1% Triton and incubated in a bleaching solution of 3% H2O2 + 3% formamide in 0.2X SSC. Embryos were then blocked in PBS + 0.1% Triton + 0.1% BSA + 5% NGS for 3 h. The blocking solution was then replaced, including primary antibodies as follows: rabbit anti-laminin (Sigma, L9393) at 1:50, rabbit anti-PhH3 (Abcam, ab5176) at 1:500, mouse anti-acetylated tubulin (Sigma, T6793) at 1:250. Primary antibody incubation was performed overnight at 4°C, followed by washes in PBS + 0.1% Triton + 0.1% BSA and then by a secondary block of PBS + 0.1% Triton + 0.1% BSA + 5% NGS for 3 h. Finally, this was replenished, to also include goat anti-rabbit and/or goat anti-mouse secondary antibodies at 1:250 and DAPI at 1:500 for overnight incubations. Rhodamine phalloidin (Thermofisher, R415) staining was performed overnight during secondary antibody incubation at 1:200 dilution. Embryos were washed thoroughly with PBS + 0.1% Triton. Imaging was performed on an Olympus V3000 inverted confocal microscope at 30X optical magnification.

### *In situ* Hybridization Chain Reaction on Embryos

Hybridization chain reaction (HCR) version 3 was performed on embryos as described in Andrews et al. (2020). Briefly, embryos were rehydrated in NPBS + 0.1% Triton X, incubated for 30 min in bleaching solution and permeabilised in 1% DMSO, 1% Triton for 3 h. They were incubated in Hybridisation Buffer (HB, Molecular Instruments) for 2 h and then probes were added in HB overnight at 37°C. The following day probe excess was removed with Wash Buffer (Molecular Instrument). Embryos were washed in 5X-SSC + 0.1% Triton X, incubated in Amplification Buffer (AB, Molecular Instruments) for 30 min and then left overnight in the dark at room temperature in AB + 0.03 μM of each hairpin (Molecular Instruments). The next day embryos were washed in the dark in 5X-SSC + 0.1% Triton X and incubated overnight with 1 μg/mL DAPI in NPBT, then washed in NPBT and transferred in a glass-bottomed dish in 100% glycerol. Imaging was performed on an Olympus V3000 inverted confocal microscope.

Probes were generated using the following sequences from the *B. lanceolatum* transcriptome (Marlétaz et al., 2018): *Six3/6* (BL08388), *SerT* (BL96109), *VGlut* (BL22589), *Otp* (BL13404), *FoxD* (BL10518).

### Image Processing

To overcome EdU signal saturation from the endoderm, nuclear EdU labelling was masked using a binary mask of the DAPI channel in Fiji. Neurons and axonal projections were manually segmented using the Surface tool of the Imaris software (IMARIS 9.7.2, Bitplane, Oxford Instruments).

## Results

### Cell Division Restricts to Two Polarised Domains During Neural Tube Morphogenesis

**FIGURE 1:**
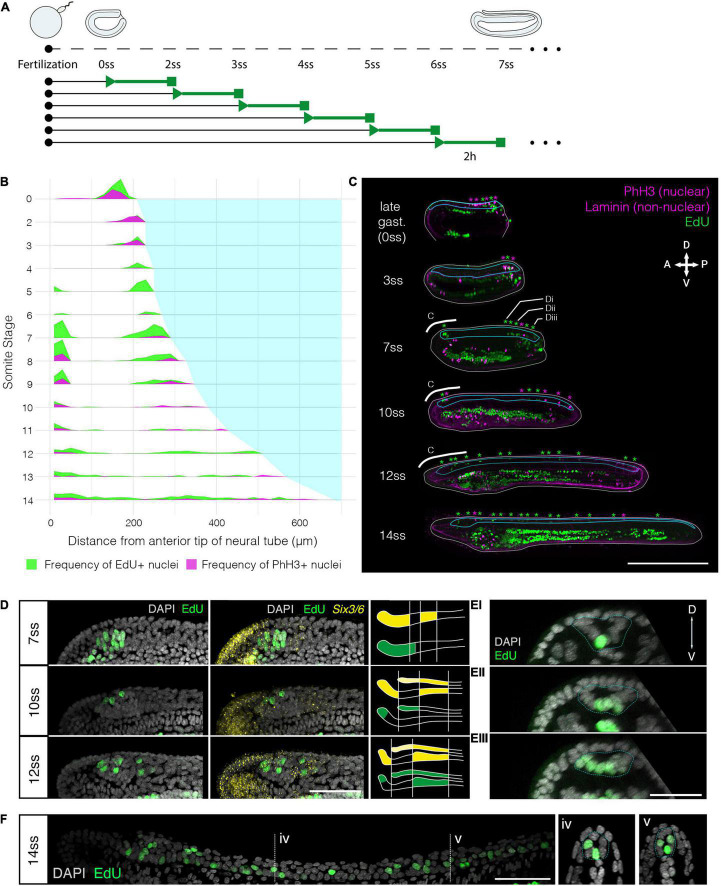
Cell division restricts to two polarised mitotic domains during neural tube morphogenesis. **(A)** Experimental design of EdU pulses experiments. Two hour EdU pulses are represented in green, while squares indicate time of fixation. The same approach was taken every 2 h for embryos up to the 14 ss stage. **(B)** Proliferation landscape for the neural tube, showing mean frequency of EdU-positive (EdU exposure for 2-h before fixation) and PhH3-positive nuclei in 20 μm bins of the anteroposterior axis at successive somite stages. “0-somite stage” refers to late gastrula 2-h before onset of somitogenesis. *n* = 114 embryos. **(C)** Mid-sagittal sections for representative embryos included in panel **(A)**, showing distribution of EdU-positive and PhH3-positive nuclei in each tissue of the body axis, which are delineated by laminin immunostaining. White lines outline the whole embryo, light blue lines outline the neural tube. Asterisks mark location of EdU/PhH3-positive nuclei using the same colour code. Scale bar is 150 μm. **(D)** Lateral z-projections of the cerebral vesicle at 7, 10, and 12 somite stages, at the level indicated by thick white lines in panel **(C)**, showing EdU incorporation and co-localisation with *Six3/6*, using HCR *in situ* hybridisation. Scale bar is 50 μm. Brain patterns are schematically represented and summarised on the right side of the panel in the same colour code. **(E)** Serial transverse sections through a 7-somite stage embryo at the posterior positions illustrated in panels **(C,Ei–iii)**, showing the EdU detection profile. Neural tube is outlined with a dashed blue line. Scale bar is 30 μm. **(F)** Lateral projection of 14-somite stage embryo, with representative transverse sections at the indicated positions: iv and v, where the neural tube is outlined with a light blue dashed line. Scale bar is 50 μm.

To define the pattern of cell division during amphioxus neural tube formation, we first constructed a spatiotemporal map of cell cycle progression between blastopore closure (0-somite stage; 2 h prior to formation of the first somite) and the early larva stage (14-somite stage) (Figure 1 and Supplementary Figure 1). At each somite-stage across this time course, we labelled embryos with markers for nuclei in two cell cycle phases. We incubated embryos in EdU for 2-h prior to fixation, detecting cells passing through S-phase, and immunostained for phosphorylated histone 3 (PhH3), to mark mitotic nuclei at the time of fixation (Figure 1A). To synthesise data across multiple specimens, we calculated the mean frequency of labelled nuclei in evenly sized bins of the anteroposterior axis at each stage, and plotted these values across stages and mean axial lengths to generate a proliferation “landscape.” This map of cell cycle progression revealed cell division in the neural tube to pass through multiple phases, exhibiting unique spatial dynamics (Figure 1B). At first, cell division occurs specifically in the extreme posterior neural plate, where it interfaces with the posterior axial mesoderm at the dorsal blastopore lip (Figure 1B, 0–4 ss, 1C 0 + 3 ss). Second, between 5 ss and 10 ss, we observed a burst of cell division restricted to the extreme anterior and posterior tips of the neural tube (Figure 1B, 5–10 ss, 1C 7 + 10 ss). At these stages, we identified almost no cell division in the central part of the neural tube, between these polarised mitotic domains. Finally, after 10 ss, cell division resumed at a low level across the entire anteroposterior axis (Figure 1B, 10–14 ss, 1C 12 + 14 ss).

Having identified a spatial restriction of cell division to the anterior and posterior poles of the neural tube, we next sought to define the pattern of cell division in each domain in finer detail. For the anterior domain, we combined EdU detection with *Six3/6* profiling by HCR *in situ*. *Six3/6* i*s* a known marker of anterior neuroectoderm with well-known boundaries in the amphioxus brain, therefore representing an ideal co-stain to contextualise the distribution of proliferating cells during development within this domain (Kozmik et al., 2007; Steinmetz et al., 2010; Range, 2014). Furthermore, *Six3/6* has been shown to regulate cell cycle in the anterior neural plate of vertebrates (Gestri et al., 2005). As previously reported, *Six3/6* expression resolves the amphioxus cerebral vesicle into three areas throughout neurulation: anterior and posterior (post-infundibular) *Six3/6*-positive domains, separated by a small region devoid of *Six3/6* expression at pre-infundibular level, what we term here as intercalated *Six3/6* negative region (Kozmik et al., 2007; Albuixech-Crespo et al., 2017). Our analysis showed that each of these areas proliferates at different time points during neural tube morphogenesis. At 7 ss, EdU labelled a relatively large group of cells in the anterior *Six3/6*-positive and intercalated Six3/6 negative region, whereas no EdU-positive cells were found in the posterior *Six3/6-*positive cluster (Figure 1D). At 10 ss, cell division declined, such that only 3–5 EdU-positive cells were identified in the anterior tip or dorsal region of the developing cerebral vesicle, within the anterior *Six3/6*-positive cluster (Figure 1D). At 12 ss, the number of EdU-positive cells in the anterior-dorsal cerebral vesicle increased, and proliferating cells became also visible in Figure 1D.

To characterise the spatial distribution of cell division in the posterior body, we analysed the pattern of EdU-positive nuclei in serial transverse sections from whole mount images along the anteroposterior axis. This was performed at 7 ss, when cell division is occurring at its greatest magnitude in the posterior neural tube, and EdU-positive are seen extending anteriorly from the chordoneural hinge (Figure 1E). At 7 ss, the posterior neural plate has not yet folded to form a tube and exhibits a “U” shape in transverse section. The most anterior EdU-positive cells in the neural tube were invariably located ventrally, in the prospective floor plate (Figure 1Ei). However, in more posterior sections, EdU-positive cells were more dispersed across the mediolateral axis of the neural plate, such that multiple EdU-positive cells were observed in each transverse section (Figure 1Eii). In sections immediately anterior to the chordoneural hinge, EdU-positive cells were found throughout the neural plate, with no conspicuous spatial bias across the mediolateral axis (Figure 1Eiii). Considered together, cell division occurs broadly in the posterior neural plate during axial elongation, but restricts to the most medial-ventral cells at more anterior axial positions.

At 14 ss, when cell division has resumed in the area between the two proliferative domains, we found that EdU labelled cells were located only in the floor plate or in medio-lateral cells, clearly visible in cross-sections of the neural tube (Figure 1F). In sum, this analysis reveals that cell division restricts during axial elongation to two polarised mitotic domains; the anterior cerebral vesicle, where different regions of the prospective brain exhibit different proliferative rates, and the posterior neural tube, where proliferative cells extend anteriorly from the chordoneural hinge into the posterior floor plate.

### Proliferation Specifically Contributes to Maturation of the Brain and Posterior Floor Plate

**FIGURE 2:**
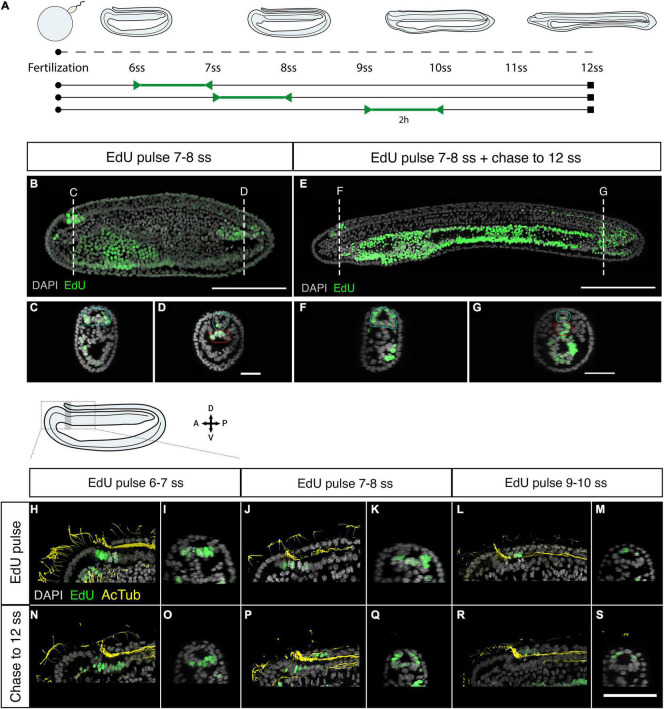
Fate of proliferating cells tracked using EdU-pulse chases. **(A)** Experimental design of Edu pulse-chase experiments. Two-hour EdU pulses are represented in green, after which embryos were raised (chase) to 12 ss and then fixed (square). **(B)** Detection of EdU-pulse between 7–8 ss with cross section details shown for the anterior **(C)**, and the posterior **(D)** neural proliferative domains. **(E)** Chase of an embryo pulsed at 7–8 ss, as shown in panel **(B)**, to 12 ss revealing that while anterior EdU-positive cells remain in the brain **(F)** posterior positive cells spread through one-third of the posterior floor plate **(G)**. In cross sections the blue circle highlights the neural tissue while the red circle defines the chordoneural hinge **(D)** and the notochord **(G)**. Scale bar is 100 μm for lateral views and 30 μm for cross-sections. **(H,I)** EdU pulses at 6–7 ss marked ventral brain cells, as shown in lateral **(H)** and sagittal **(I)** planes, which remained ventral in the cerebral vesicle of 12 ss embryos, as shown in lateral **(N)** and sagittal **(O)** planes of the brain. **(J–M)** At later stages lateral and dorsal proliferating cells in the brain, as shown by Edu pulses between 7–8 ss [Panel **(J)**: lateral plane; **(K)** sagittal plane] and 9–10 ss [Panel **(L)**: lateral plane; **(M)** sagittal plane] contributed to progressively dorsal cell populations, as shown in embryos at 12 ss in lateral **(P,R)** and sagittal **(Q,S)** planes. EdU in green, acetylated tubulin in yellow. Scale bar is 50 μm.

We next sought to map the contributions of proliferative cells in the anterior and posterior mitotic domains of the neural tube to tissue morphogenesis. We employed a pulse-chase approach to mark proliferative cells in each domain when the polarised cell division dynamic is conspicuous (5–10 ss) and map the distribution of their clones later in development (Figure 2A). Pulses of EdU at 7–8 ss labelled distinct clusters of cells at the anterior tip of the neural tube (Figures 2B,C), and a single-file row of cells in the posterior neural tube extending anteriorly from the chordoneural hinge of the tailbud (Figures 2B,D). The ventral endoderm was also strongly labelled but was non-overlapping with the two neural proliferative domains. After the EdU pulse, EdU was washed out extensively with fresh sea water, and embryos were left to develop until the 12 ss. At 12 ss (Figure 2A), we found that EdU-positive cells in the anterior CNS remained confined to the cerebral vesicle, and did not spread posteriorly into the rest of the neural tube (Figures 2E,F). Meanwhile, EdU-positive cells in the posterior neural tube contributed to 1/3*^rd^* of the floor plate (Figures 2E,G). Furthermore, some EdU-positive cells initially located in the chordoneural hinge at 7–8 ss, were found in the posterior notochord at 12 ss (Figure 2G compare with Figure 2D).

As we showed above, neural progenitors within the cerebral vesicle proliferate at different time points between 7 and 12 ss. We therefore performed pulse-chase experiments with higher temporal resolution to determine how cell division contributes to the maturation of specific brain areas during development. We combined EdU detection with acetylated tubulin immunostaining, which marks the cilia of the neural canal, to distinguish dorsal and ventral areas within the cerebral vesicle. Clones of cells that incorporated EdU between 6 and 7 ss remained ventral at 12 ss, meaning they contribute broadly to the ventral side of the cerebral vesicle along its anteroposterior axis (Figures 2H,I,N,O). In contrast, EdU-positive cells labelled in the anterior neural plate between 7 and 8 ss were positioned more laterally, and therefore contributed most significantly to the dorso-lateral walls of the cerebral vesicle after neurulation (Figures 2J,K,P,Q). Finally, those labelled between 9 and 10 ss contributed exclusively to the dorsal side of the cerebral vesicle (Figures 2L,M,R,S). Considered together, these data suggest that cell division in the brain is spatially restricted, increasing cell numbers locally in a ventral-to-dorsal temporal sequence. Meanwhile, proliferative cells in the posterior neural tube and chordoneural hinge specifically contribute to elongation of the posterior floor plate and become broadly dispersed across the anteroposterior axis within the posterior body.

#### Cell Division Is Dispensable for Broad Body Plan Patterning but Necessary for Proper Axial Tissue Geometry

**FIGURE 3:**
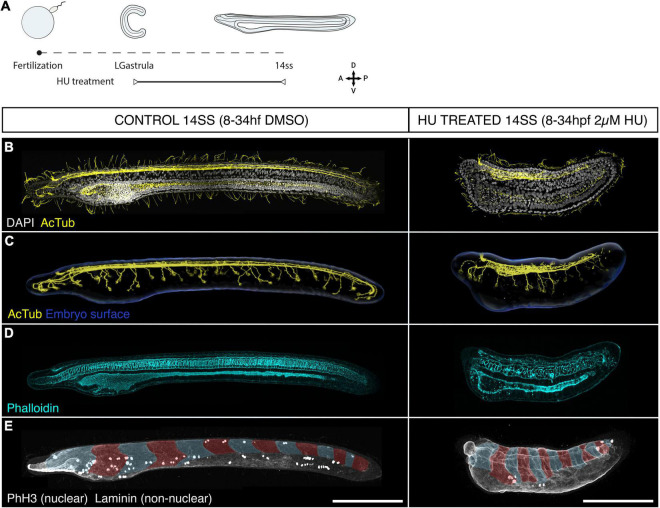
Cell division is dispensable for body plan patterning but required for establishing a normal geometry. **(A)** Experimental design of hydroxyurea (HU) treatments. **(B–E)** Control embryos (treated with DMSO) and embryos treated with 2 μM HU are shown side by side in lateral z-projections. **(B)** The general morphology of these embryos is shown through immunostaining with acetylated tubulin (cilia and axonal scaffold) and DAPI (nuclei). **(C)** The structure of the axonal scaffold is shown by segmentation of the acetilated tubulin channel from embryos in panel **(A)**. **(D)** Phalloidin (actin) staining in the same embryos reveals the presence of a notochord and a ventral endoderm. **(E)** Immunostaining with laminin and PhH3 reveals the presence of an equal number of somites in both control and treated embryos (false-colouredin blue and read overlay), and the efficacy of the treatment to arrest proliferation, which results in a significant loss of PHH3-positive nuclei in HU-treated embryos. Scale bars are 150 μm for both control and treated embryos. HU-treated phenotype representative of *n* = 13 embryos imaged.

After tracing the distribution and fates of proliferative cells in the amphioxus neural tube, we sought to assess the functional contributions of cell division to neural tube morphogenesis. To this end, we inhibited cell division from mid-gastrulation (8 hpf), when cell division is widespread throughout the embryo (Holland and Holland, 2006), to the early larva stage (14 ss) using hydroxyurea (HU) to arrest DNA synthesis (Figure 3A).

Surprisingly, HU-treated embryos remained viable until the 14 ss stage, hatching and swimming normally (data not shown) despite the lack of cell division (Figure 3). While prolonged exposure to HU resulted in widespread cell death, indicated by pyknotic DAPI-stained nuclei at the 14 ss (Supplementary Figure 2A), we found that the major patterns of the body plan were intact. The neural tube was present and internalised beneath the surface ectoderm (Figures 3B,C). Immunostaining for acetylated-tubulin in both control and treated embryos showed a central neural canal and axonal tracts running on the ventral side of the neural tube (Figures 3B,C). The neural tube of HU-treated embryos showed a clearly defined cerebral vesicle, which opened anteriorly through the neuropore and had rostral axonal projections similar to DMSO-treated control embryos. Furthermore, peripheral epidermal sensory neurons formed normally along the body axis and projected to the neural tube, although the size of each neuron was visibly larger than in control embryos (Figure 3C). These observations demonstrate the developmental robustness of the nervous system anlage in amphioxus, which forms even in the absence of proliferation during early neurogenesis (Figure 3C).

Aside from the nervous system, the notochord was present at the axial midline in HU-treated embryos, with a characteristic stack-of-coins pattern, and a complete pattern of somites was formed within the paraxial mesoderm on the left and right sides of the embryo (Figures 3D,E). While this means that the amphioxus body plan is robust to the loss of cell division, our results also show that the geometry of the embryos is severely distorted. Most conspicuously, HU-treated embryos failed to elongate, reaching less than half the AP length of DMSO-treated siblings by 14 ss. Our results thus indicate that proliferation may be dispensable at the stages treated to form the body plan, in terms of tissue composition and topology, but it is key to confer specific body traits with proper geometry.

### Anterior Proliferation Is Required for Cell Type Diversity of the Brain

Having found that cell division is dispensable for the formation of the neural tube, we next investigated its role within each proliferative domain. Given that the amphioxus brain is relatively diverse in cell types (Benito-Gutiérrez, 2006; Albuixech-Crespo et al., 2017), we next focussed on the brain and investigated the role of cell-division in cell type diversification.

**FIGURE 4:**
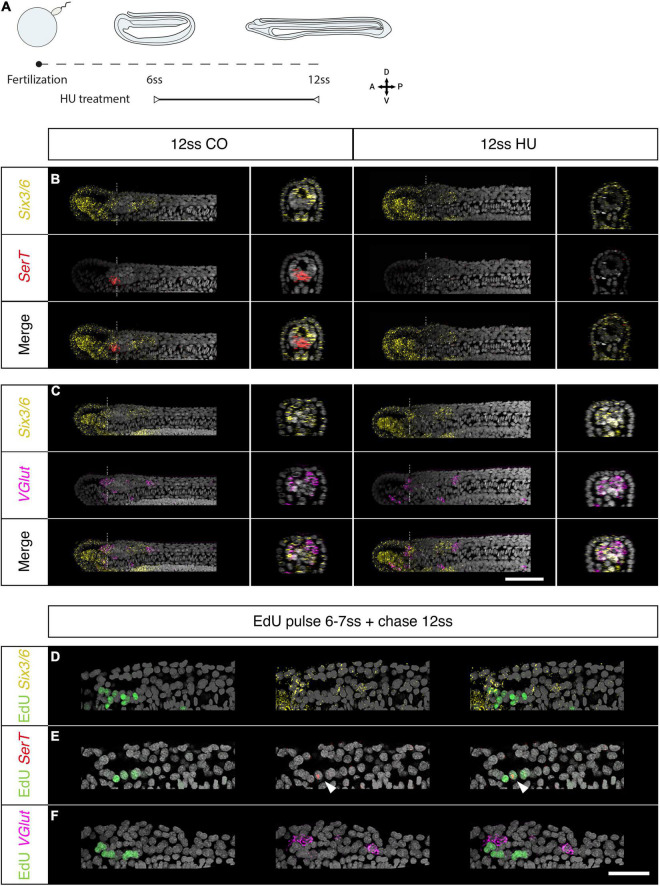
Brain proliferation is required to establish the neuronal type repertoire in the amphioxus larva. **(A–C)** Expression of neurotransmitter markers in control and Hydroxyurea (HU)-treated embryos at 12 ss through HCR. **(A)** Experimental design of HU treatments. **(B)** *SerT* expression in control embryos is localised at the border of the anterior *Six3/6* domain but disappears following HU treatment. **(C)** In the neural tube, *VGlut* is expressed in anterior-most cells within the anterior *Six3/6* domain and caudal to the posterior *Six3/6* domain. This pattern remains the same in HU-treated embryos. Scalebar is 50 μm. **(D–F)** Brain expression of neurotransmitter markers in embryos pulsed with EdU at 6–7 ss and chased to 12 ss. **(D)** EdU is detected in a few cells within the anterior *Six3/6* domain. **(E)** *SerT*-positive neurons are labelled with EdU. **(F)** *VGlut* expression is not detected in EdU-positive cells. Scalebar is 30 μm. *Six3/6* in yellow, *SerT* in red, *VGlut* in magenta, EdU in green.

**FIGURE 5:**
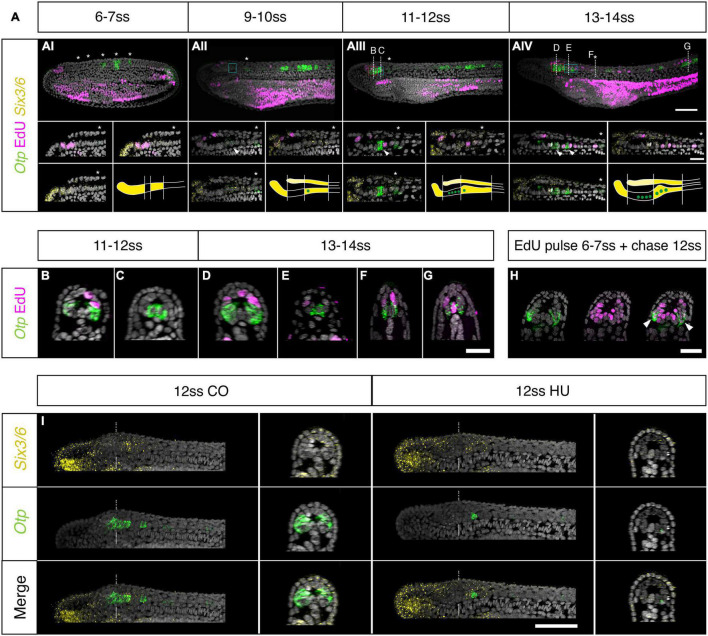
*Otp* expression and brain proliferation during neurulation. **(A)** Co-detection of *Otp* and *Six3/6* expression through HCR *in situ* hybridisation and EdU incorporation. **(Ai)** At 7 ss *Otp* is expressed in five clusters of cells in the trunk region (asterisks). Expression is strong in the posterior three clusters but very low in the anterior two clusters (asterisk in insets). **(Aii)** At 10 ss, a pair of *Otp* positive neurons appear in the posterior *Six3/*6 domain of cerebral vesicle (arrowhead in insets), but proliferation is still restricted to the anterior cerebral vesicle. **(Aiii)** At 12 ss two ventro-lateral clusters of *Otp*-positive cells are visible in the anterior cerebral vesicle. At this stage, cell division starts in the posterior cerebral vesicle; the posterior *Otp*-positive pair is not proliferating but cells adjacent to them are labelled by EdU (insets). **(Aiv)** At 14 ss, the number of posterior *Otp*-positive neurons increases (arrowhead). Asterisks in all insets show the first *Otp* cluster in the trunk. Patterns of *Six3/6* and *Otp* co-expression are schematically representated at the bottom of each panel for every developmental stage. Scale bar is 50 μm; 20 μm for insets. **(B–G)** Cross section of 12 and 14 ss embryos showing the anterior ventro-lateral **(B,D)**, posterior medial **(C,E)**, and trunk **(F,G)** *Otp* cells. Scalebar: 20 μm **(H)** 12 ss embryos EdU-pulsed at 7 ss show co-localization of EdU and *Otp* (arrowheads) at the level of D in panel **(Aiv)**. **(I)** Inhibition of cell division with 2 μM hydroxyurea leads to a loss of the ventro-lateral *Otp* clusters in the *Six3/6*-negative domain, while the posterior medial *Otp* cells are not affected. Scale bar is 50 μm. EdU in magenta, *Six3/6* in yellow, *Otp* in green.

To this aim we arrested cell-division for a shorter interval of time, starting at the 6 ss stage, when proliferation is specifically localised in the brain and the chordoneural hinge, and raised these embryos up to the 12 and 14 ss stages (Figure 4A and Supplementary Figure 2B). We then examined the expression of genes known to mark specific cell types in chordate brains: the serotonergic marker *SerT*, the glutamatergic marker *VGlut* and the transcription factors *Six3/6* and *Otp* (Figures 4, 5, and Supplementary Figures 2C–E). The treated embryos did not show any changes in *Six3/6* expression, which showed the typical tripartite arrangement as in controls, also reported previously by Kozmik et al. (2007) (Figures 4B,C). This observation was consistent with these domains forming prior to axial elongation and before the anterior cerebral vesicle starts proliferating, at stages before HU was administered. By contrast, the treated embryos were devoid of the small cluster of serotonergic (*SerT-*positive) neurons that form Row2 in the cerebral vesicle of 12 ss embryos and contribute to the frontal eye complex (Candiani et al., 2012; Vopalensky et al., 2012). Our results therefore suggest that localised proliferation is needed to generate this entire row of serotonergic neurons (Figure 4A and Supplementary Figure 2C). On the contrary, we did not see any differences on the expression of the glutamate transporter *VGlut*, which localised, as in control embryos, in the anterior *Six3/6*-positive domain, and caudally to the posterior *Six3/6*-positive cluster in 12 and 14 ss embryos, in similar positions to those previously reported (Candiani et al., 2012; Figure 4B and Supplementary Figure 2D).

Overall, our results suggest that some cell types in the amphioxus brain are dependent on particular progenitors proliferating at specific times. With this hypothesis in mind, we next pulsed embryos with EdU between the 6 and 7 ss stage and raised these embryos up to the 12 ss stage, in order to re-examine the expression of *Six3/6*, *SerT*, and *VGlut* at the same stages above. We found that while the majority of *Six3/6* cells are Edu-negative, a few cells double *Six3/6*-Edu positive located at the most anterior end of the cerebral vesicle (Figure 4D). As we suspected, the few *SerT*-positive detectable at this stage are all Edu-positive, indicating that they derive from progenitors proliferating at 6 ss (Figure 4E). Conversely, none of the glutamatergic neurons marked by *VGlut* were EdU-positive, confirming that these cells were born earlier in development, before the 6 ss stage (Figure 4F).

To investigate the possible role of cell-division in brain regionalisation, we additionally investigated the expression of *Otp*, which in vertebrates is expressed in the hypothalamus, diencephalon and hindbrain, where it contributes to specify a variety of neuronal cell types (Del Giacco et al., 2008; Fernandes et al., 2013). At the 6 ss stage, *Otp* is expressed in the trunk region of the nervous system, as previously reported (Albuixech-Crespo et al., 2017; Figure 5Ai). However, at later stages, we find that *Otp* is progressively upregulated in the anterior CNS. First, at 10 ss, in a pair of ventro-medially located cells in the posterior *Six3/6*-positive region, at a post-infundibular level (Figure 5Aii. See Figures 5D,E for later stages of this population). Second, at 12 ss, in two prominent ventro-lateral clusters located in the brain, at pre-infundibular level, in the intercalated *Six3/6*-negative region (Figures 5Aiii,B. See Figure 5D for a later stage of this population). Edu co-detection in pulsed embryos before fixation showed no double *Otp*-Edu positive cells (Figure 5A), indicating that *Otp* cells are not proliferating at these stages.

As we found here, progenitors proliferating at the 6–7 ss mostly contribute to ventral parts of the cerebral vesicle (See Figure 2). Given this, we set out to investigate whether any of these progenitors could have given rise to the brain *Otp* clusters first observed in the pre-infundibular regions at 12 ss. We next probed 12 ss embryos that had been treated with HU from the 6–7 ss stage, and found that the ventro-lateral clusters of *Otp*- neurons in the intercalated *Six3/6*-negative pre-infundibular region were lost in these embryos (Figure 5I). The post-infundibular ventro-medial Otp clusters, were not affected by the treatment (Figure 5I). However, we noticed, that treated embryos raised up to the 14 ss had less cells in their post-infundibular ventro-medial cluster respect from controls (Supplementary Figure 2E), suggesting that the intermingling Edu-positive cells detected at 12 and 14 ss (See insets in Figures 5Aiii,iv) might be contributing to amplify the post-infundibular *Otp* cluster, at these stages of development. These results also suggested that the ventro-lateral cluster of *Otp* cells in the brain might have been originated by a localised burst of proliferation at the 6–7 ss. To test this, we again pulsed embryos with Edu at the 6–7 ss and raised these embryos up to the 12 ss. Co-localisation of Edu and *Otp* in these embryos demonstrated that cells in the *Otp* ventro-lateral cluster in the brain were born at the 6–7 ss and differentiated later, at the 12 ss (Figure 5H).

The expression of *Otp* here reported, highlights that the amphioxus brain continues to regionalise during neurulation via local addition of new cells. To investigate how these new areas of expression fit into the current proposed scheme of brain regionalisation (Albuixech-Crespo et al., 2017), we co-profiled the expression of *Six3/6*, *Otp* and *FoxD* at 7 and 14 ss (Supplementary Figure 3). Taking the 7 ss stage as a reference, and *Six3/6* and *FoxD* to bridge developmental differences in gene expression, we found that at later stages *FoxD* is downregulated from the rostral *Six3/6* domain and instead remains strongly expressed within the intercalated *Six3/6* negative region, at pre-infundibular level, in a position fully overlapping with the anterior *Otp* ventrolateral clusters in the brain (Supplementary Figures 3A–D). These results suggest that the pre-infundibular *Otp* domain intercalates at the level of the intermediate-caudal HyPth, forming a separated hypothalamic region (indicated as Hyp in Supplementary Figure 3F), posteriorly bordered by the *Six3/6-Otp* positive post-infundibular domain, which would correspond to the region proposed as DiMes by Albuixech-Crespo and collaborators (Supplementary Figures 3G–H).

Considered together, these data indicate that cell division timely regulates the specification of particular brain cell types at distinct time points, thereby incrementing the neural cell type diversity throughout development and therefore contributing to regionalise the brain.

### Posterior Midline Progenitors Have a Common Origin in the Dorsal Blastopore Lip

Having identified a role for cell division in generating cell type diversity in the brain, we next focussed on its contribution in the posterior neural tube, where we found cell division extends the floor plate. Fate mapping studies in vertebrates have revealed axial progenitor cells residing in the chordoneural hinge of the tailbud to derive from the dorsal blastopore lip of the late gastrula, or its equivalent in amniotes, the node-streak border (Selleck and Stern, 1991; Gont et al., 1993; Catala et al., 1996; Cambrey and Wilson, 2007). During axial elongation, a subset of these axial progenitors clonally expands, and contributes new cells to the posterior notochord and floor plate. We have previously shown that proliferation starting at 6 ss is required for full elongation of the amphioxus body axis (Andrews et al., 2021). However, while posterior axial progenitors have been hypothesised in amphioxus, their diversity and dynamics have not yet been defined experimentally (Hatschek, 1893; Lwoff, 1894; Schubert et al., 2001). We therefore sought to achieve this by tracing chordoneural hinge progenitors to their site of origin in the gastrula.

**FIGURE 6:**
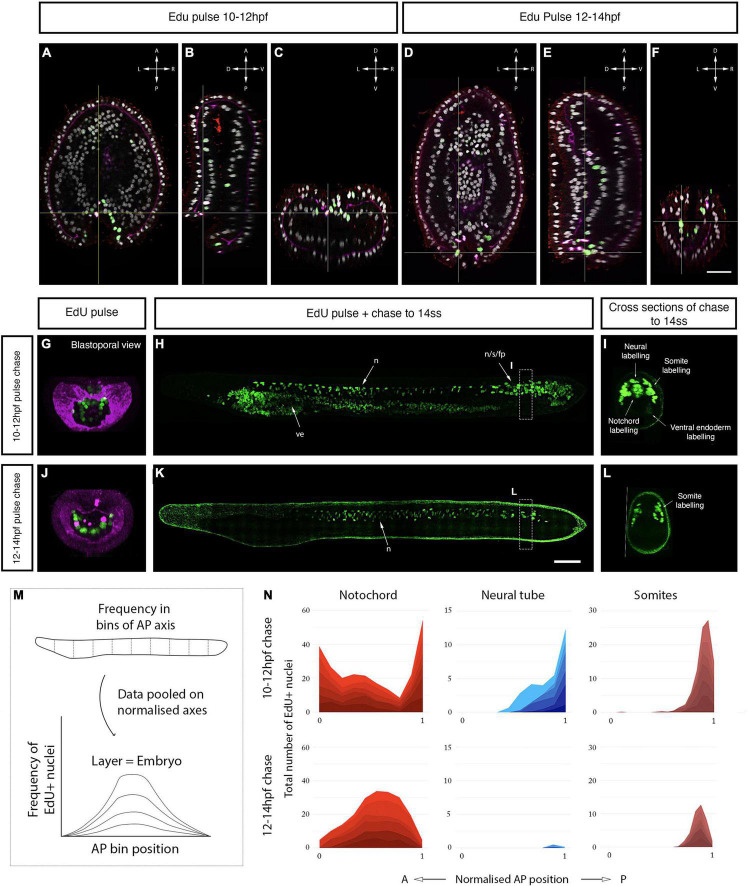
Axial progenitor fates are spatially regionalised across the late blastopore lip. **(A–C)** Distribution of EdU- and PhH3-positive nuclei after an EdU pulse between 10 and 12 hpf, shown in orthogonal coronal **(A)**, sagittal **(B)**, and transverse sections **(C)** intersecting at the level of the blastopore (as indicated by the yellow lines). Scale bars are 30 μm. **(D–F)** Distribution of EdU-positive nuclei after an EdU pulse between 12 and 14 hpf, shown in a coronal **(D)**, sagittal **(E)**, and transverse sections **(F)** intersecting at the level of the blastopore (as indicated by the yellow lines). Compass shows orientation of each plane in panels **(A–F)**. **(G)** Maximum projection through the blastopore in an embryo pulsed with EdU between 10 and 12 hpf, as shown in panel **(C)**, marking proliferative cells in the dorsal and upper-lateral lips. **(H)** Maximum projection through the sagittal midline of an embryo EdU pulsed at 10–12 hpf, as shown in panel **(G)**, showing contribution of EdU-positive cells in the dorsal blastopore lip to neural tube, notochord and somites. **(I)** Transverse section through posterior body of embryo shown in panel **(H)**, showing tissue-specificity of EdU-positive cells. **(J)** Maximum projection through the blastopore in an embryo pulsed with EdU 12–14 hpf, as shown in panel **(F)**, showing proliferating cells in the ventral and lower-lateral lips. **(K)** Maximum projection through the sagittal midline of an embryo EdU pulsed at 12–14 hpf, as shown in panel **(J)**, revealing the contribution of proliferating cells to the posterior somites. **(L)** Transverse section through posterior body of embryo shown in panel **(K)**, showing tissue-specificity of EdU-positive cells. Scalebars are 50 μm. **(M)** Schematic of approach used to quantify EdU labelling in pulse-chased specimens as a frequency curve across normalised AP length, following quantification in 10 evenly-sized bins. **(N)** Stacked area graphs for each pulse-chase condition and tissue. *n* = 6 embryos per condition. EdU in green, PhH3 (nuclear) and laminin in magenta, acetylated tubulin in red. fp, floor plate; s, somites; n, notochord; ve, ventral endoderm.

Since direct cell marking and tracking *in vivo* is technically challenging in amphioxus, we further took advantage of the highly localised proliferation dynamics we identified here to spatially map the origin of chordoneural hinge progenitors in the gastrula, prior to axial elongation. Closer observation of EdU incorporation in the blastopore of the late gastrula revealed a temporal delay in cell cycle progression across the dorsoventral axis: In EdU pulses between 10 and 12 hpf (1–2 ss), EdU was primarily incorporated by cells in the dorsal and upper-lateral lips of the blastopore (Figures 6A–C,G). In contrast, EdU pulses between 12 and 14 hpf (3–4 ss) marked cells in the ventral and lower-lateral lips of the blastopore (Figures 6D–F,J). Based on this observation, we reasoned that we could determine the derivatives of each blastopore subdomain by performing successive pulse-chase experiments, following the sequential enrichment of EdU across the dorsoventral axis of the blastopore.

In the first of these pulse-chase experiments (EdU pulse 10–12 hpf), we found that cells initially located in the dorsal and upper-lateral blastopore lips at 12 hpf made broad contributions to the posterior body when chased to 14 ss (Figure 6H). At 14 ss, EdU-positive cells were enriched in the chordoneural hinge of the tailbud, and extended anteriorly from it to populate the posterior floor plate, notochord and posterior 4–5 somites (Figures 6H,I). This is shown qualitatively in midline sections of representative 14 ss embryos (Figure 6H), and quantitatively by calculating the frequency of EdU-positive nuclei in evenly-sized bins of the anteroposterior axis in each axial tissue (Figures 6M,N). Labelling of the chordoneural hinge, floor plate and notochord in this experiment recapitulated the distribution we noted previously in a pulse-chase experiment with EdU incorporation at 7–8 ss when the chordoneural hinge is proliferating (Figure 2C). This finding therefore suggested that progenitor cells of the chordoneural hinge have an origin in the blastopore lip of the late gastrula.

In the second pulse-chase experiment (EdU pulse 12–14 hpf), we found that cells initially located in the ventral and lower-lateral blastopore lips of the late gastrula (Figure 6J) gave rise to posterior somites at 14 ss, with almost no EdU-positive cells detected in the posterior notochord or floor plate (Figures 6K,L). In this second pulse-chase experiment, loss of EdU labelling in the dorsal blastopore lip during the EdU pulse (compare Figure 6G and Figure 6J) correlated with a loss of labelling in the posterior notochord and floor plate at 14 ss and reduced density of labelling in the posterior somites (compare Figures 6I,L and Figure 6N). By taking advantage of temporal changes in blastopore proliferation dynamics, this experiment therefore indicated a spatial regionalisation of progenitor subtypes in the late blastopore lip, which includes: (a) a dorsal lip containing progenitors that relocate to the chordoneural hinge of the tailbud and enter a proliferative phase after 6 ss to elongate the posterior floor plate and notochord; and (b) lateral and ventral lips containing progenitors that give rise to cells populating the posterior 4–5 somites, which do not contribute to the chordoneural hinge population.

## Discussion

Proliferation is a key contributor to morphological complexity in nervous system development, with demonstrated functions in the regulation of tissue size, geometry, and cellular complexity. As such, the number of cell divisions in neural progenitors and the balance between proliferation and differentiation exhibits striking variation between species (Kriegstein et al., 2006; Fish et al., 2008; Montgomery, 2017; Logan et al., 2018; Briscoe and Ragsdale, 2019). While vertebrate species are characterised by large brains, including anatomically distinct compartments and complex stratification of neural cell types, the two groups of invertebrate chordates–cephalochordates and tunicates–possess simpler central nervous systems, composed of fewer cells and exhibiting limited morphological regionalisation (Sasakura et al., 2012; Holland and Holland, 2021). A key question is what role cell division plays in the morphogenesis of these simple nervous systems, and whether these dynamics may predict the emergence of more complex properties in vertebrates.

### Landscapes of Cell Division in Amphioxus Show Developmental Modularity During the Formation of the Central Nervous System

**FIGURE 7:**
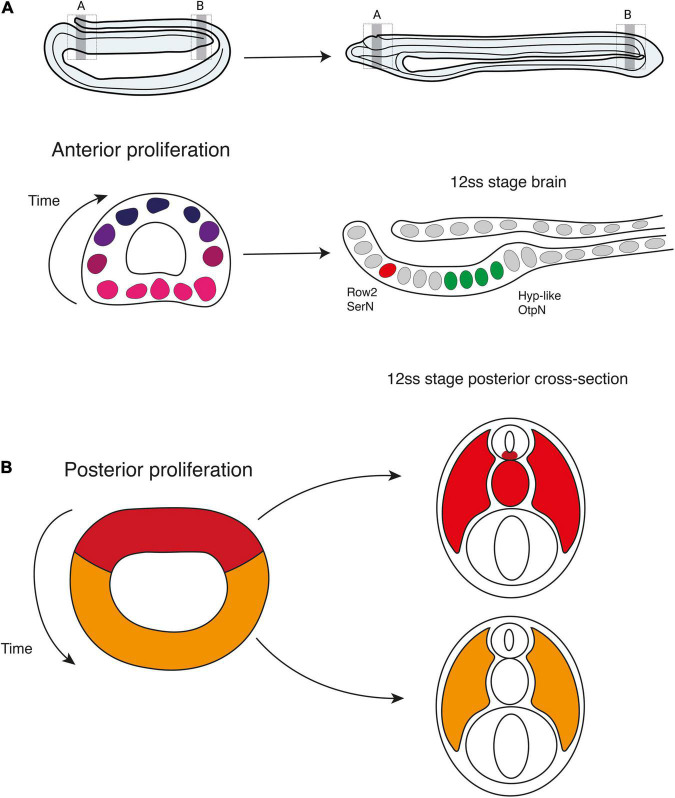
Contributions of cell proliferation to nervous system and axial development. During neurulation, cell division is restricted to the anterior and posterior poles of the neural plate. **(A)** Anteriorly, the brain develops following a ventral to dorsal proliferative gradient. Ventral proliferation is required to specify Row2 serotonergic neurons of the frontal eye and hypothalamic-like *Otp*-positive neurons. **(B)** Posteriorly, early proliferating cells in the dorsal blastoporal region contribute to the floor plate, notochord, and somites, while progenitors in the ventral and lateral regions of the blastopore divide later and are incorporated into the posterior somites.

In this study, we provide new insight into the distribution and function of cell division in the embryonic nervous system of amphioxus. We expand on previous studies (Holland and Holland, 2006; Andrews et al., 2021) by constructing a detailed spatiotemporal map of cell cycle progression in the neural tube, which highlights a sudden polarisation of cell division to the anterior and posterior tips of the nascent nervous system at the mid-neurula stage, concomitant with elongation of the anteroposterior axis. This includes discrete mitotic domains in the presumptive cerebral vesicle, and the chordoneural hinge of the tailbud, extending anteriorly into the posterior floor plate. By marking and following these cells in pulse-chase experiments, we demonstrate that anterior proliferative cells locally increase cell number within the cerebral vesicle in a ventral-to-dorsal temporal sequence, and that this wave of cell division is a pre-requisite for the emergence of brain cell type diversity (Figure 7A). In contrast, we show that proliferative cells in the chordoneural hinge and posterior neural plate generate floor plate progenitors that extend across 1/3rd of the anteroposterior axis. We further show that these posterior progenitors can be traced back to the dorsal blastopore lip of the gastrula, where they lie adjacent to posterior somite progenitors (Figure 7B). Finally, we find that the polarised cell division dynamic collapses prior to the onset of larval life, when proliferation resumes throughout the nervous system.

The pattern of cell division we describe in amphioxus differs from what is seen in the vertebrate CNS in that the neuroectoderm of vertebrates proliferates extensively throughout its development. Early neural progenitors (neuroepithelial and radial glial cells) are known to undergo symmetrical divisions across the anteroposterior length of the neural plate and early neural tube (Qian et al., 2000; Götz and Huttner, 2005; Yingling et al., 2008). These divisions are used to expand the repertoire of stem cells that will later give rise to neurons and glia (Qian et al., 2000; Noctor et al., 2004). In fact, the rate of proliferation of these cells directly affects the size and regionalisation of adult mammalian brains. For example, disruption of early signalling (e.g., FGF and Wnt/Bcatenin) controlling neural tube proliferation in mammals deeply affects the size, thickness and convolution of the brain (Vaccarino et al., 1999; Chenn and Walsh, 2002; Megason and McMahon, 2002). In contrast, in other invertebrate chordates like ascidians, cells have been observed proliferating in the larval sensory vesicle and, to a lesser extent, in the visceral ganglion, thereby resembling more closely the anterior proliferation we observed in amphioxus. However, proliferation is absent posteriorly in the ascidian neural tube, indicating that neural morphology in these animals is primarily driven by cell re-arrangement and cell shape change (Tarallo and Sordino, 2004; Nakayama et al., 2005). This diversity of proliferative strategies in the different chordate subphyla supports the idea that tweaks in the timing and magnitude of cell division have played important roles in the emergence of neural complexity during chordate evolution.

Interestingly, we observed that proliferation in the trunk neural tube at 14 ss occurs specifically in cells of the floor plate and medio-lateral cells. Both cell types have recently been described as having a glial-like molecular signature in the early larva (Bozzo et al., 2021). Bozzo and collaborators speculate that proliferation of medio-lateral cells expressing the glial marker EAAT2 results in the formation of ependymal and ependymoglial cells in late larva stages (Lacalli and Kelly, 2002). Our results here provide experimental support for this hypothesis: while ventro-lateral cells already express neural markers, such as neurotransmitter synthesis and transport proteins (Candiani et al., 2012) and member of the adenosine deaminase acting on RNA (ADAR) family with function in RNA-editing (Zawisza-álvarez et al., 2020), medio-lateral cells are still dividing at the beginning of the larval stage and could therefore represent progenitors that maintain a glial signature and have a supporting role in the larval neural tube. Supporting this idea, we find that the transcription factor *Otp* is expressed in ventro-lateral cells of the trunk neural tube that are not proliferating at the early larva stage (Figures 5F,G).

### Localised Proliferation in the Amphioxus Brain Is Key for the Proper Formation of the Frontal Eye and Hypothalamus

Anterior proliferative cells remain confined to the amphioxus cerebral vesicle, but cell division occurs sequentially in a ventral-to-dorsal direction, thereby contributing to different areas of the brain at distinct time points in development. This pattern suggested that the proliferation dynamics might have a role in regionalising the amphioxus brain, by contributing with new cells at specific times and sites to be fated to particular types. Our targetted cell-division arrest experiments demonstrated this to be the case.

We found that localised proliferation in the amphioxus anterior brain is necessary to generate the anterior serotonergic cells of the frontal eye complex, as we previously proposed (Benito-Gutiérrez et al., 2018). These neurons are born from progenitors proliferating between the 6 and 7 ss stage. Upon pulsing with Edu at this precise stage, we manage to retain the Edu label in the daughter cells, which we found differentiating at the 12 ss into a serotonergic fate. Preventing cell-division at the 6–7 ss stage eliminates the entire population and so these embryos have a frontal eye complex lacking in serotonergic neurons. Having found that not all the cell types of the frontal eye complex are dependent on this localised burst of proliferation at 6–7 ss (e.g., glutamatergic cell), our results indicate that proliferation in the brain does not act homogeneously to extend the brain anteriorly, as previously proposed (Holland and Holland, 2006). Instead, proliferation is intercalated and essential to diversify the cell type repertoire of the frontal eye complex.

Such intercalated growth is also illustrated by the development of the pre-infundibular brain region expressing *Otp*. Here we show that the entire pre-infundibular *Otp* domain depends on the localised proliferation of precursors located ventrally at the prime meridian of the cerebral vesicle (half-way between the frontal eye and the infundibular organ) at the 6–7 ss (Figure 5H). We identify these precursors through Edu pulse and chase experiments, and we defined them as fated to become ventrolateral *Otp* cells by arresting cell division, as in treated embryos this brain region is completely missing. These ventro-lateral *Otp* cells could correspond to previously described neurons with very small apices that appear unusually crowded in front of the infundibular organ (Lacalli, 2000). *Otp* cells elsewhere in the neural tube and at the post-infundibular region are born earlier in development, before the 6–7 ss. However, we find that arresting cell division until the 14 ss reduces the number of cells in the ventro-medial *Otp* cluster located in the post-infundibular region, indicating that some of the intermingled cells that we see proliferating between the 12 and 14 ss might be contributing with new cells to this cluster.

Interestingly, *Otp* is key to the development of the hypothalamic neuroendocrine system in all vertebrates (Wang and Lufkin, 2000; Del Giacco et al., 2008), and in ascidians, *Otp* cells have been shown to localise in a region identified as hypothalamic, and surrounded by *Six3/6*-positive cells (Moret et al., 2005). In amphioxus, we further show that the pre-infundibular population of *Otp* is expressed in the same region as the hypothalamic marker *FoxD* (Bedont et al., 2015). Altogether this suggests that the pre-infundibular *Six3/6-/FoxD* + /*Otp* + region could correspond to part of the amphioxus hypothalamus, while the post-infundibular *Six3/6* + /*FoxD*-/*Otp* + region could be contributing to diencephalic structures (for further detail in the context of previously proposed brain regionalisation schemes See Supplementary Note in Supplementary Material). This might indicate that amphioxus posseses a seemingly continuous hypothalamic field uninterrupted with the eye field, as described in fish, axolotl, frogs, and chicken (Staudt and Houart, 2007).

Overall, our results reveal a putative pre-infundibular hypothalamic region within the amphioxus brain that develops through intercalated proliferation and growth from the 6–7 ss, and a post-infundibular area which, albeit gastrulation-derived, augments cell number from the 12 ss stage.

### The Amphioxus Tailbud Is a Source of Posterior Floor Plate and Notochord Cells

In the posterior body, we found using EdU pulse-chase analysis that proliferative cells in the chordoneural hinge of the tailbud specifically contribute to the elongation of the floor plate and notochord. This is mediated by a burst of cell division occurring in the mid-neurula after 6 ss, when proliferation restricts to the most posterior and anterior ends of the neural tube. Prior to this developmental stage, cells proliferate in the posterior neural plate throughout axial development, and pass sequentially through S-phase and mitosis. This means that the posterior neural plate is an active proliferative domain from gastrulation to the end of somitogenesis. Given this, we were able to track the progeny of dividing axial progenitors in the blastopore via a series of EdU pulse-chase experiments. With this approach, we demonstrate that midline axial progenitors originate from the dorsal blastopore lip of the late gastrula. There, they lie adjacent to progenitors of the posterior somites, which are located in the lateral and ventral blastopore lips.

As such, the location and fates of these axial progenitor cells in amphioxus bears strong similarity to the dynamics of midline progenitor cells identified in vertebrates. In amniotes, homotopic grafting approaches and direct cell marking have located midline progenitors of the floor plate and notochord that arise in the node-streak border of the gastrula, and undergo clonal expansion within the chordoneural hinge (Catala et al., 1996; Cambray and Wilson, 2002; Mugele et al., 2018). Similarly, fate mapping of the dorsal blastopore lip in *Xenopus* has located derivatives in the posterior notochord in floor plate (Gont et al., 1993). In all cases, midline progenitor cells further extend the tissue primordia formed during gastrulation and do so to a degree that is species-specific, aligning with variations in nutritional supply (O’Farrell, 2015; Steventon et al., 2016). In externally developing anamniotes, axial progenitors undergo little clonal expansion and make minor contributions to axial length, whereas in amniotes they undergo extensive clonal expansion and growth, thereby driving a significant posterior enlargement of the embryo (Steventon et al., 2016; Attardi et al., 2019). Our results in amphioxus therefore support a model in which midline axial progenitors may be an ancestral trait in chordate embryogenesis, in which evolutionary transitions in the magnitude of cell division, and therefore the sizes of axial progenitor clones, have facilitated divergent contributions to axial length.

## Conclusion

Our work provides a spatiotemporal map of cell cycle progression during the early development of amphioxus (up to the early larval stage). Proliferation is widespread in the archenteron throughout the whole period observed. However, by focussing our analysis on cell division within the CNS, we find that the nervous system is spatially segregated in three different developmental modules characterised by distinct cell division dynamics. Anteriorly, within the brain, proliferation is required locally to increase the neural type repertoire at specific time points. We find that cell proliferation is necessary to fully equip the frontal eye with the correct neurotransmitting cells, before the eye spot fully develops into a functional photoreceptor able to integrate sensory information (Vopalensky et al., 2012). In the brain, proliferation is also needed to grow the ventral portion of the brain, which we identify here as hypothalamic based on *Otp* expression. It is tempting to speculate that this phase of hypothalamic development is preparatory for entering the subsequent feeding phase, when a neuroendocrine system would need to be in place. In parallel, neuronal cells gradually differentiate in the trunk nervous system, independently of cell division, and posteriorly, tailbud progenitors contribute to elongating the posterior neural axis, which at the period analysed remains generally undifferentiated. Unexpectedly, the amphioxus body plan is robust to cell cycle arrest. Embryos develop with fewer cells but they still harbour a dorsal neural tube with an underlying notochord and the correct number of somites, demonstrating that cell division is primarily required to confer a proper geometry and to increase neuronal type complexity.

## Data Availability Statement

The original contributions presented in the study are included in the article/Supplementary Material, further inquiries can be directed to the corresponding author.

## Author Contributions

GG and TA: acquisition and analysis of data and writing—original draft. ÈB-G: project administration. All authors study design, manuscript review and editing, and read and agreed to the published version of the manuscript.

## Conflict of Interest

The authors declare that the research was conducted in the absence of any commercial or financial relationships that could be construed as a potential conflict of interest.

## Publisher’s Note

All claims expressed in this article are solely those of the authors and do not necessarily represent those of their affiliated organizations, or those of the publisher, the editors and the reviewers. Any product that may be evaluated in this article, or claim that may be made by its manufacturer, is not guaranteed or endorsed by the publisher.

## References

[B1] Albuixech-Crespo B. López-Blanch L. Burguera D. Maeso I. Sánchez-Arrones L. Moreno-Bravo J. A. (2017). Molecular regionalization of the developing amphioxus neural tube challenges major partitions of the vertebrate. brain. *PLoS Biol.* 15:e2001573. 10.1371/journal.pbio.2001573 28422959 PMC5396861

[B2] Allaway K. C. Muñoz W. Tremblay R. Sherer M. Herron J. Rudy B. (2020). Cellular birthdate predicts laminar and regional cholinergic projection topography in the forebrain. *Elife* 9:e63249. 10.7554/ELIFE.63249 33355093 PMC7758062

[B3] Ando H. Sato T. Ito T. Yamamoto J. Sakamoto S. Nitta N. (2019). Cereblon control of zebrafish brain size by regulation of neural stem cell proliferation. *iScience* 15 95–108. 10.1016/j.isci.2019.04.007 31055217 PMC6501120

[B4] Andrews T. G. Gattoni G. Busby L. Schwimmer M. A. Benito-Gutiérrez È (2020). “Hybridization chain reaction for quantitative and multiplex imaging of gene expression in amphioxus embryos and adult tissues,” in *In Situ Hybridization Protocols*, eds Nielsen B. S. Jones J. (Berlin: Springer Nature), 179–194. 10.1007/978-1-0716-0623-0 PMC7612682 32394382

[B5] Andrews T. G. R. Pönisch W. Paluch E. Steventon B. J. Benito-Gutiérrez È (2021). Single-cell morphometrics reveals ancestral principles of notochord development. *Development* 148:dev199430. 10.1242/dev.199430 34343262 PMC8406538

[B6] Attardi A. Fulton T. Florescu M. Shah G. Muresan L. Lenz M. O. (2019). Neuromesodermal progenitors are a conserved source of spinal cord with divergent growth dynamics. *Development* 146:dev175620. 10.1242/dev.175620 30333213 PMC6240315

[B7] Bedont J. L. Newman E. A. Blackshaw S. (2015). Patterning, specification, and differentiation in the developing hypothalamus. *Wiley Interdiscip. Rev. Dev. Biol.* 4 445–468. 10.1002/wdev.187 25820448 PMC5890958

[B8] Benito-Gutiérrez E. Stemmer M. Rohr S. D. Schumacher L. N. Tang J. Marconi A. (2018). Patterning of a telencephalon-like region in the adult brain of amphioxus. *bioRxiv* [preprint]. 10.1101/307629

[B9] Benito-Gutiérrez È (2006). A gene catalogue of the amphioxus nervous system. *Int. J. Biol. Sci.* 2 149–160. 10.7150/ijbs.2.149 16763675 PMC1474150

[B10] Benito-Gutiérrez È. (2011). “Amphioxus as a model for mechanisms in vertebrate development,” in *eLS* (Chichester: John Wiley & Sons, Ltd), 1–12. 10.1002/9780470015902.a0021773

[B11] Benito-Gutiérrez È Gattoni G. Stemmer M. Rohr S. D. Schuhmacher L. N. Tang J. (2021). The dorsoanterior brain of adult amphioxus shares similarities in expression profile and neuronal composition with the vertebrate telencephalon. *BMC Biol.* 19:110. 10.1186/s12915-021-01045-w 34020648 PMC8139002

[B12] Benito-Gutiérrez È Weber H. Bryant D. V. Arendt D. (2013). Methods for generating year-round access to Amphioxus in the laboratory. *PLoS One* 8:e71599. 10.1371/journal.pone.0071599 23990962 PMC3753313

[B13] Bozzo M. Lacalli T. C. Obino V. Caicci F. Marcenaro E. Bachetti T. (2021). Amphioxus neuroglia: Molecular characterization and evidence for early compartmentalization of the developing nerve cord. *Glia* 69 1654–1678. 10.1002/glia.23982 33624886

[B14] Briscoe S. D. Ragsdale C. W. (2019). Evolution of the chordate telencephalon. *Curr. Biol.* 29 R647–R662. 10.1016/j.cub.2019.05.026 31287987 PMC11073819

[B15] Cambray N. Wilson V. (2002). Axial progenitors with extensive potency are localised to the mouse chordoneural hinge. *Development* 129 4855–4866. 10.1242/dev.129.20.4855 12361976

[B16] Cambrey N. Wilson V. (2007). Two distinct sources for a population of maturing axial progenitors. *Development* 134, 2829–2840. 10.1242/dev.02877 17611225

[B17] Candiani S. Moronti L. Ramoino P. Schubert M. Pestarino M. (2012). A neurochemical map of the developing amphioxus nervous system. *BMC Neurosci.* 13:59. 10.1186/1471-2202-13-59 22676056 PMC3484041

[B18] Catala M. Teillet M. De Robertis EM. Le Douarin ML. (1996). A spinal cord fate map in the avian embryo: while regressing, Hensen’s node lays down the notochord and floor plate thus joining the spinal cord lateral walls. *Development* 122 2599–2610. 10.1242/dev.122.9.2599 8787735

[B19] Caviness V. S. Takahashi T. Nowakowski R. S. (1995). Numbers, time and neocortical neuronogenesis: a general developmental and evolutionary model. *Trends Neurosci.* 18 379–383. 10.1016/0166-2236(95)93933-O 7482802

[B20] Chenn A. Walsh C. A. (2002). Regulation of cerebral cortical size by control of cell cycle exit in neural precursors. *Science* 297 365–369. 10.1126/science.1074192 12130776

[B21] Del Giacco L. Pistocchi A. Cotelli F. Fortunato A. E. Sordino P. (2008). A peek inside the neurosecretory brain through Orthopedia lenses. *Dev. Dyn.* 237 2295–2303. 10.1002/dvdy.21668 18729222

[B22] Fernandes A. M. Beddows E. Filippi A. Driever W. (2013). Orthopedia transcription factor otpa and otpb paralogous genes function during dopaminergic and neuroendocrine cell specification in larval zebrafish. *PLoS One* 8:e75002. 10.1371/journal.pone.0075002 24073233 PMC3779234

[B23] Fish J. L. Dehay C. Kennedy H. Huttner W. B. (2008). Making bigger brains - The evolution of neural-progenitor-cell division. *J. Cell Sci.* 121 2783–2793. 10.1242/jcs.023465 18716282

[B24] Garcia-Fernàndez J. Benito-Gutiérrez È (2009). It’s a long way from amphioxus: Descendants of the earliest chordate. *BioEssays* 31 665–675. 10.1002/bies.200800110 19408244

[B25] Gestri G. Carl M. Appolloni I. Wilson S. W. Barsacchi G. Andreazzoli M. (2005). Six3 functions in anterior neural plate specification by promoting cell proliferation and inhibiting Bmp4 expression. *Development* 132 2401–2413. 10.1242/dev.01814 15843413 PMC2789257

[B26] Gont L. K. Steinbeisser H. Blumberg B. De Robertis E. M. (1993). Tail formation as a continuation of gastrulation: The multiple cell populations of the Xenopus tailbud derive from the late blastopore lip. *Development* 119 991–1004. 10.1242/dev.119.4.991 7916680

[B27] Götz M. Huttner W. B. (2005). The cell biology of neurogenesis. *Nat. Rev. Mol. Cell Biol.* 6 777–788. 10.1038/nrm1739 16314867

[B28] Hatschek B. (1893). *The Amphioxus and its Development*. London: Swan Sonnenschein & Co.

[B29] Holland L. Z. (2017). “Invertebrate origins of vertebrate nervous systems,” in *Evolution of Nervous Systems*, (Amsterdam: Elsevier), 3–23. 10.1016/B978-0-12-804042-3.00001-4

[B30] Holland L. Z. Holland N. D. (2021). *Cephalochordates: A window into vertebrate origins*. 1st ed. Amsterdam: Elsevier Inc, 10.1016/bs.ctdb.2020.07.001 33602486

[B31] Holland N. D. (2016). Nervous systems and scenarios for the invertebrate-to-vertebrate transition. *Philos. Trans. R. Soc. B Biol. Sci.* 371:20150047. 10.1098/rstb.2015.0047 26598728 PMC4685584

[B32] Holland N. D. Holland L. Z. (2006). Stage- and tissue-specific patterns of cell division in embryonic and larval tissues of amphioxus during normal development. *Evol. Dev.* 8 142–149. 10.1111/j.1525-142x.2006.00085.x 16509893

[B33] Huttner W. B. Kosodo Y. (2005). Symmetric versus asymmetric cell division during neurogenesis in the developing vertebrate central nervous system. *Curr. Opin. Cell Biol.* 17 648–657. 10.1016/j.ceb.2005.10.005 16243506

[B34] Kozmik Z. Holland N. D. Kreslova J. Oliveri D. Schubert M. Jonasova K. (2007). Pax–Six–Eya–Dach network during amphioxus development: Conservation in vitro but context specificity in vivo. *Dev. Biol.* 306 143–159. 10.1016/j.ydbio.2007.03.009 17477914

[B35] Kriegstein A. Noctor S. Martínez-Cerdeño V. (2006). Patterns of neural stem and progenitor cell division may underlie evolutionary cortical expansion. *Nat. Rev. Neurosci.* 7 883–890. Available online at: www.nature.com/reviews/neuro 10.1038/nrn2008 17033683

[B36] Lacalli T. C. (1996). Frontal eye circuitry, rostral sensory pathways and brain organization in amphioxus larvae: Evidence from 3D reconstructions. *Philos. Trans. R. Soc. B Biol. Sci.* 351 243–263. 10.1098/rstb.1996.0022

[B37] Lacalli T. C. (2000). Cell morphology in amphioxus nerve cord may reflect the time course of cell differentiation. *Int. J. Dev. Biol.* 44 903–906. 10.1387/ijdb.11206331 11206331

[B38] Lacalli T. C. (2021). Innovation through heterochrony: an amphioxus perspective on telencephalon origin and function. *Front. Ecol. Evol.* 9:666722. 10.3389/fevo.2021.666722

[B39] Lacalli T. C. Holland N. D. West J. E. (1994). Landmarks in the anterior central nervous system of amphioxus larvae. *Philos. Trans. R. Soc. London. Ser. B Biol. Sci.* 344 165–185. 10.1098/rstb.1994.0059

[B40] Lacalli T. C. Kelly S. J. (2002). Floor plate, glia and other support cells in the anterior nerve cord of amphioxus larvae. *Acta Zool.* 83 87–98. 10.1046/j.1463-6395.2002.00101.x

[B41] Logan C. J. Avin S. Boogert N. Buskell A. Cross F. R. Currie A. (2018). Beyond brain size: Uncovering the neural correlates of behavioral and cognitive specialization. *Comp. Cogn. Behav. Rev.* 13 55–90. 10.3819/CCBR.2018.130008

[B42] Lwoff B. (1894). *Die Bildung der Primären Keimblätter und Die Entstehung der Chorda und des Meso- Derms bei den Wirbelthieren*. Moskau: Gedruckt in der Universitätsbuchdruckerei.

[B43] Marlétaz F. Firbas P. N. Maeso I. Tena J. J. Bogdanovic O. Perry M. (2018). Amphioxus functional genomics and the origins of vertebrate gene regulation. *Nature* 564 64–70. 10.1038/s41586-018-0734-6 30464347 PMC6292497

[B44] Megason S. G. McMahon A. P. (2002). A mitogen gradient of dorsal midline Wnts organizes growth in the CNS. *Development* 129 2087–2098. 10.1242/dev.129.9.2087 11959819

[B45] Montgomery S. H. (2017). “Evolution of large brain and body size in mammals,” in *Evolution of Nervous Systems: Second Edition*, (Amsterdam: Elsevier), 103–136. 10.1016/B978-0-12-804042-3.00034-8

[B46] Moret F. Christiaen L. Deyts C. Blin M. Vernier P. Joly J. S. (2005). Regulatory gene expressions in the ascidian ventral sensory vesicle: Evolutionary relationships with the vertebrate hypothalamus. *Dev. Biol.* 277 567–579. 10.1016/j.ydbio.2004.11.004 15617694

[B47] Mugele D. Moulding D. A. Savery D. Molè M. A. Greene N. D. E. Martinez-Barbera J. P. (2018). Genetic approaches in mice demonstrate that neuro-mesodermal progenitors express T/Brachyury but not Sox2. *bioRxiv* [Preprint]. 10.1101/503854

[B48] Nakayama A. Satoh N. Sasakura Y. (2005). Tissue-specific profile of DNA replication in the swimming larvae of Ciona intestinalis. *Zoolog. Sci.* 22 301–309. 10.2108/zsj.22.301 15795492

[B49] Nieuwenhuys R. (2017). Principles of current vertebrate neuromorphology. *Brain. Behav. Evol.* 90 117–130. 10.1159/000460237 28988231

[B50] Noctor S. C. Martinez-Cerdeño V. Ivic L. Kriegstein A. R. (2004). Cortical neurons arise in symmetric and asymmetric division zones and migrate through specific phases. *Nat. Neurosci.* 7 136–144. 10.1038/nn1172 14703572

[B51] O’Farrell P. H. (2015). Growing an embryo from a single cell: A hurdle in animal life. *Cold Spring Harb. Perspect. Biol.* 7:a019042. 10.1101/cshperspect.a019042 26254311 PMC4632664

[B52] Qian X. Shen Q. Goderie S. K. He W. Capela A. Davis A. A. (2000). Timing of CNS cell generation. *Neuron* 28 69–80. 10.1016/s0896-6273(00)00086-6 11086984

[B53] Rakic P. (1995). A small step for the cell, a giant leap for mankind: a hypothesis of neocortical expansion during evolution. *Trends Neurosci.* 18 383–388. 10.1016/0166-2236(95)93934-P 7482803

[B54] Range R. (2014). Specification and positioning of the anterior neuroectoderm in deuterostome embryos. *Genesis* 52 222–234. 10.1002/dvg.22759 24549984

[B55] Sasakura Y. Mita K. Ogura Y. Horie T. (2012). Ascidians as excellent chordate models for studying the development of the nervous system during embryogenesis and metamorphosis. *Dev. Growth Differ.* 54 420–437. 10.1111/j.1440-169X.2012.01343.x 22524611

[B56] Schubert M. Holland L. Z. Stokes M. D. Holland N. D. (2001). Three amphioxus Wnt genes (AmphiWnt3, AmphiWnt5, and AmphiWnt6) associated with the tail bud: the evolution of somitogenesis in chordates. *Dev. Biol.* 240, 262–273. 10.1006/dbio.2001.046 11784062

[B57] Selleck M. A. J. Stern C. D. (1991). Fate mapping and cell lineage analysis of Hensen’s node in the chick embryo. *Development* 112, 615–626. 10.1242/dev.112.2.615 1794328

[B58] Staudt N. Houart C. (2007). The prethalamus is established during gastrulation and influences diencephalic regionalization. *PLoS Biol.* 5:878–888. 10.1371/journal.pbio.0050069 17341136 PMC1808486

[B59] Steinmetz P. R. H. Urbach R. Posnien N. Eriksson J. Kostyuchenko R. P. Brena C. (2010). Six3 demarcates the anterior-most developing brain region in bilaterian animals. *Evodevo* 1:14. 10.1186/2041-9139-1-14 21190549 PMC3025827

[B60] Steventon B. Duarte F. Lagadec R. Mazan S. Nicolas J. F. Hirsinger E. (2016). Species-specific contribution of volumetric growth and tissue convergence to posterior body elongation in vertebrates. *Development* 143 1732–1741. 10.1242/dev.126375 26989170

[B61] Steventon B. Martinez Arias A. (2017). Evo-engineering and the cellular and molecular origins of the vertebrate spinal cord. *Dev. Biol.* 432 3–13. 10.1016/j.ydbio.2017.01.021 28192080

[B62] Tarallo R. Sordino P. (2004). Time course of programmed cell death in Ciona intestinalis in relation to mitotic activity and MAPK signaling. *Dev. Dyn.* 230 251–262. 10.1002/dvdy.20055 15162504

[B63] Temple S. Shen Q. (2013). “Cell Biology of Neuronal Progenitor Cells,” in *Patterning and Cell Type Specification in the Developing CNS and PNS*, (Amsterdam: Elsevier Inc.), 261–283. 10.1016/B978-0-12-397265-1.00076-9

[B64] Vaccarino F. M. Schwartz M. L. Raballo R. Nilsen J. Rhee J. Zhou M. (1999). Changes in cerebral cortex size are governed by fibroblast growth factor during embryogenesis. *Nat. Neurosci.* 2 246–253. 10.1038/8163 10195217

[B65] Vopalensky P. Pergner J. Liegertova M. Benito-Gutiérrez E. Arendt D. Kozmik Z. (2012). Molecular analysis of the amphioxus frontal eye unravels the evolutionary origin of the retina and pigment cells of the vertebrate eye. *Proc. Natl. Acad. Sci.* 109 15383–15388. 10.1073/pnas.1207580109 22949670 PMC3458357

[B66] Wang W. Lufkin T. (2000). The murine Otp homeobox gene plays an essential role in the specification of neuronal cell lineages in the developing hypothalamus. *Dev. Biol.* 227 432–449. 10.1006/dbio.2000.9902 11071765

[B67] Wicht H. Lacalli T. C. (2005). The nervous system of amphioxus: structure, development, and evolutionary significance. *Can. J. Zool.* 83 122–150. 10.1139/z04-163

[B68] Wilson V. Olivera-Martinez I. Storey K. G. (2009). Stem cells signals and vertebrate body axis extension. *Development* 136 1591–1604. 10.1242/dev.039172 19395637

[B69] Yingling J. Youn Y. H. Darling D. Toyo-oka K. Pramparo T. Hirotsune S. (2008). Neuroepithelial stem cell proliferation requires LIS1 for precise spindle orientation and symmetric division. *Cell* 132 474–486. 10.1016/j.cell.2008.01.026 18267077 PMC2265303

[B70] Yu J. Satou Y. Holland N. D. Shin-i T. Kohara Y. Satoh N. (2007). Axial patterning in cephalochordates and the evolution of the organizer. *Nature* 44 613–617. 10.1038/nature05472 17237766

[B71] Zawisza-álvarez M. Pérez-Calles C. Gattoni G. Garcia-Fernàndez J. Benito-Gutiérrez È Herrera-úbeda C. (2020). The ADAR family in amphioxus: RNA editing and conserved orthologous site predictions. *Genes* 11:1440. 10.3390/genes11121440 33265998 PMC7761149

