# Supplementary material for: Restricted Proliferation During Neurogenesis Contributes to Regionalisation of the Amphioxus Nervous System
Source: Front Neurosci. 2022 Mar 24;16:812223. doi: 10.3389/fnins.2022.812223 (PMC8987370; doi:10.3389/fnins.2022.812223)
Supplement: Supplementary file 1 [file Presentation_1.pdf]

Supplementary Material

1 Supplementary Figures:

1.1 Supplementary Figure1:

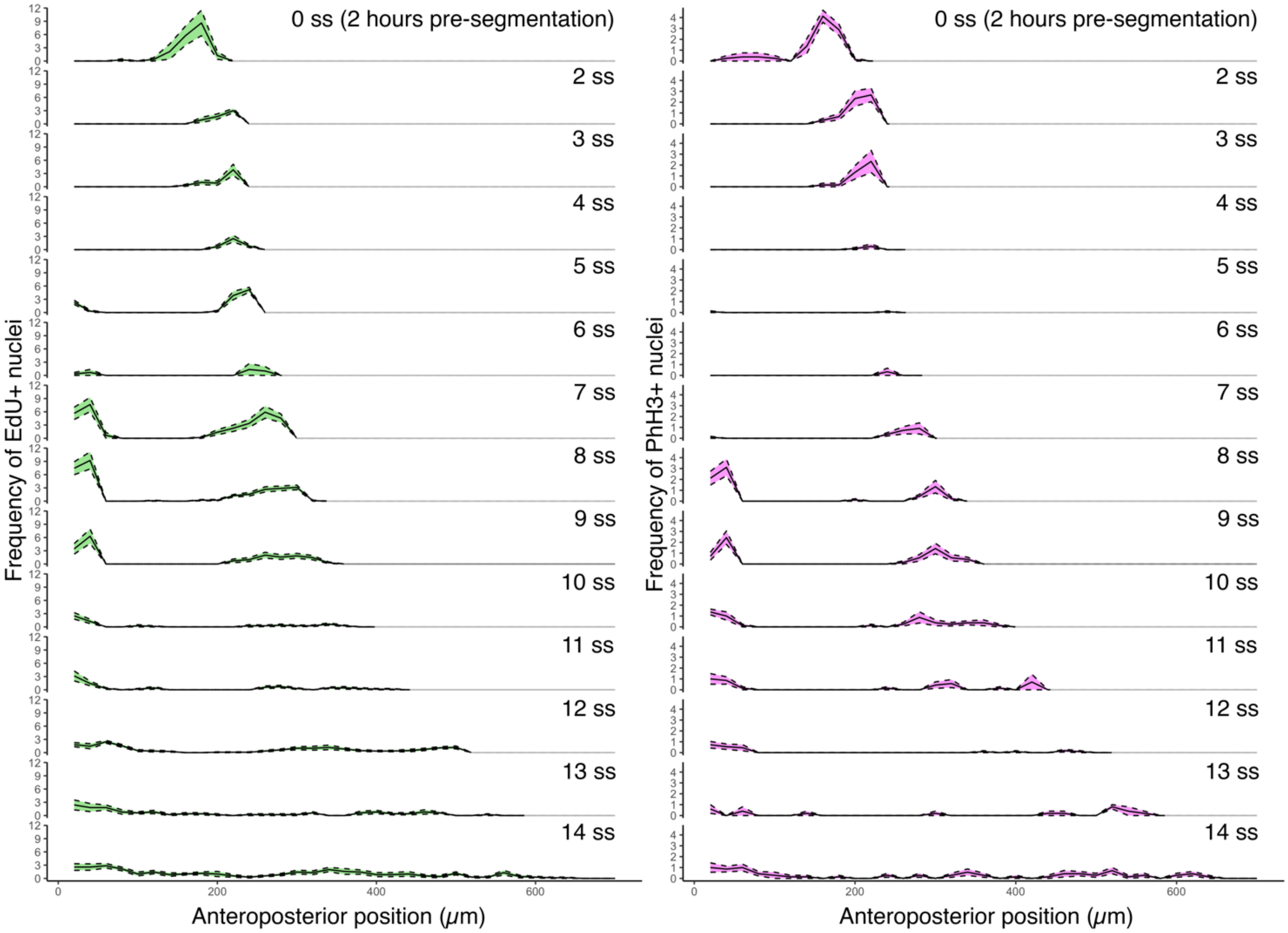

Extended Data: Neural tube proliferation landscapes. Mean frequency of EdU+ (left) and PhH3+ (right) nuclei across the anteroposterior axis of the neural tube, measured for each somite stage between 0 ss (2-hours prior to onset of somitogenesis) and 14 ss. Mean frequency of labelled nuclei was measured in 20µm bins of the anteroposterior axis, between the anterior tip of the cerebral vesicle and the chordoneural hinge of the tailbud. x axis shows normalised axial position, scaled to mean neural tube length for the given stage. Solid line shows mean frequency, dashed lines show mean +/- 1 standard error. n = 114 embryos.

1.2 Supplementary Figure 2

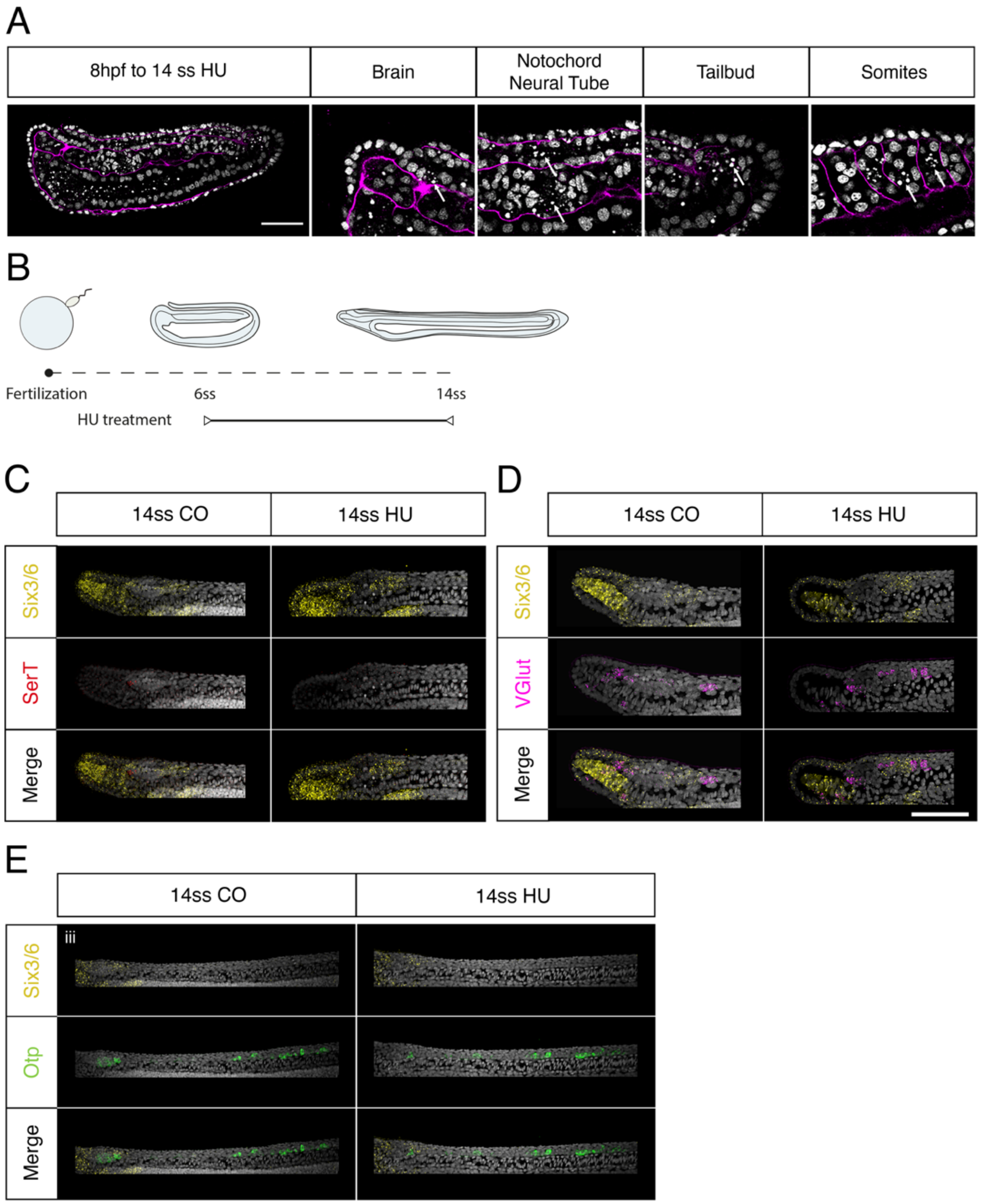

Inhibition of proliferation through hydroxyurea treatment to the 14 ss stage. (A) Visualization of pyknotic nuclei after long HU exposure (8h gastrulae – 14ss) in different compartments of the amphioxus body **(B)** Experimental design of late hydroxyurea treatments used in this study. **(C-D)** Expression of *Six3/6* (yellow) *SerT* (red) and *VGlut* (magenta) remains similar to 12ss and responds in the same way to inhibition of proliferation: *SerT* expression in the cerebral vesicle is lost while *Six3/6* and *VGlut*-positive cells persist in HU-treated embryos. **(E)** *Otp (green)* expression is lost in the anterior clusters in the *Six3/6*-negative domain, and is reduced to only a pair of cells in the posterior *Six3/6*-positive domain following HU treatment. Scale bar: 50µm.

1.3 Supplementary Figure 3

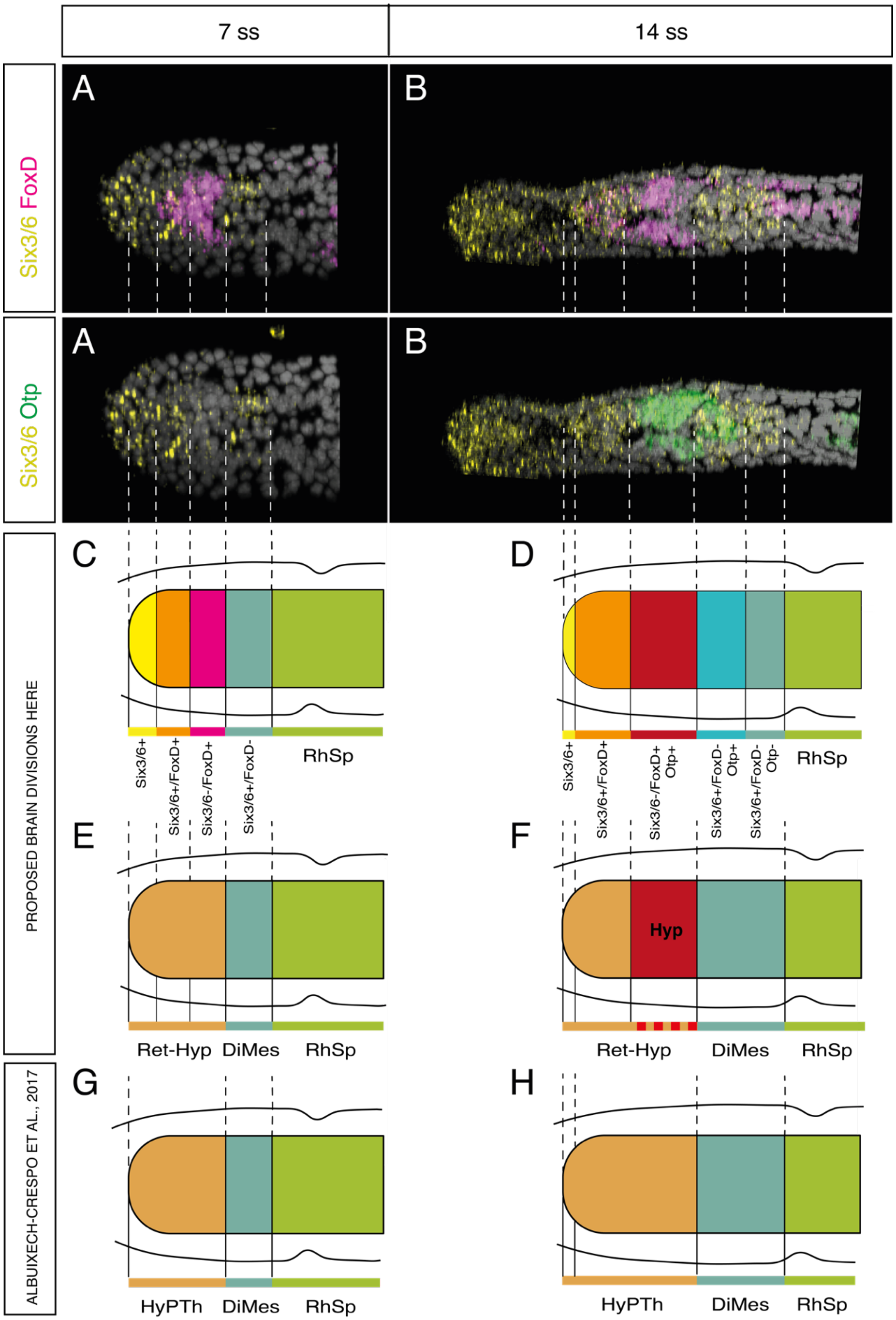

Molecular characterization of the cerebral vesicle at the 14ss stage. (A) Co-detection of *Six3/6* (yellow) and *FoxD* (magenta) at the 7 ss stage (dorsal view) shows regionalization of the neural plate consistent with previous studies (Albuixech-Crespo et al., 2017). **(B)** Co-expression of *Six3/6*, *FoxD* and *Otp* (green) at 14 ss (dorsal view) reveals regionalization of the larval brain. *FoxD* and *Otp* are both expressed in the intercalated *Six3/6*-negative region, while only *Otp* is also expressed in medial cells of the posterior *Six3/6*-positive region. **(C-D)** Schematic representation of the expression domains of the markers considered in this study at 7ss (C) and 14ss (D) (dorsal view). **(E-F)** Proposed regionalization of the amphioxus brain at 7ss (E) and 14ss (F). **(G-H)** Proposed correspondence with the model of AP brain regionalization proposed by Albuixech-Crespo et al., 2017 in 7ss (G) and 14ss (H) embryos.

2 Supplementary Note:

It has been recently proposed that the brain of early amphioxus embryos (7ss) is divided into a rostral hypothalamo-prethalamic primordium (HyPth) and a caudal diencephalic-mesencephalic primordium (DiMes) (Albuixech-Crespo et al., 2017). To identify these boundaries in our embryos, we co-profiled *Otp* with *Six3/6* and *FoxD*, which were used by Albuixech-Crespo and collaborators to set the HyPth and DiMes limits. At the same stage reported in Albuixech-Crespo et al., 2017, we find that *Six3/6* is adittionally expressed in the rostral HyPth. Otherwise, our results agree with those reported by Albuixech-Crespo and collaborators in that: *Six3/6* is absent from the intermediate and caudal HyPth (Supplementary Figure 3), a region that we term here as intercalated *Six3/6* negative region; that *FoxD* is expressed througout the rostral, intermediate and caudal HyPth; and that the DiMes division is *Six3/6+*/*FoxD-*, a region that we term here as post-infundibular *Six3/6* positive domain (Supplementary Figure 3). Later in development, at 14ss, the rostral HyPth remains *Six3/6+*/*FoxD+*, despite FoxD expression being noticeably weaker (Supplementary Figure 3). Similarly, the DiMes remains *Six3/6+*/*FoxD-*, reinforcing the idea that the post-infundibular *Otp* cluster in this region might contribute to other diencephalic structures. The region at the level of the intermediate and caudal HyPth, remains FoxD+ but enlarges considerably by addition of new cells that also form the pre-infundibular *Otp* complex (Figure 5). Given that *FoxD* and *Otp* are both necessary for hypothalamus development in vertebrates (Bedont et al., 2015), this suggests that amphioxus possesses a hypothalamic field continuous with the eye field, as described in fish, axolotl, frogs and chicken (Staudt and Houart 2007). Our results therefore suggest caution when interpreting this brain region as having a mixed pre-thalamic character. Albuixech-Crespo and collaborators proposed the pre-thalamic character in relation to the bounday between Fezf and Irx, which according to their studies is set at the DiMes boundary. Our results clearly show that, by the same definition as Six3/6+/FoxD-, the DiMes boundary aligns to the infundibular organ. Taking into consideration that, as we show here, the pre-infundibular region in amphioxus is set during neurulation, and that pre-thalamic precursors are set in vertebrates during gastrulation (Staudt and Houart 2007), it would be worth investigating if other pre-thalamic or thalamic markers are expressed at post-infundibular level where we know, following our results here, that there are gastrulation-derived precursors still differentiating.
